# Testing the effect of the Himalayan mountains as a physical barrier to gene flow in *Hippophae tibetana* Schlect. (Elaeagnaceae)

**DOI:** 10.1371/journal.pone.0172948

**Published:** 2017-05-10

**Authors:** La Qiong, Wenju Zhang, Hao Wang, Liyan Zeng, H. John B. Birks, Yang Zhong

**Affiliations:** 1 Institute of Biodiversity Science, School of Life Sciences, Fudan University, Shanghai, China; 2 Department of Biology, Tibet University, Lhasa, China; 3 Department of Biology, University of Bergen, Bergen, Norway; 4 Environmental Change Research Centre, University College London, London, United Kingdom; National Cheng Kung University, TAIWAN

## Abstract

*Hippophae tibetana* is a small, dioecious wind-pollinated shrub endemic to the Tibetan-Qinghai Plateau. It is one of the shrubs that occur at very high elevations (5250 m a.s.l.). The Himalayan mountains provides a significant geographical barrier to the Qinghai-Tibetan Plateau, dividing the Himalayan area into two regions with Nepal to the south and Tibet to the north. There is no information on how the Himalayan mountains influence gene flow and population differentiation of alpine plants. In this study, we analyzed eight nuclear microsatellite markers and cpDNA *trn*T-*trn*F regions to test the role of the Himalayan mountains as a barrier to gene flow between populations of *H*. *tibetana*. We also examined the fine-scale genetic structure within a population of *H*. *tibetana* on the north slope of Mount (Mt.) Everest. For microsatellite analyses, a total of 241 individuals were sampled from seven populations in our study area (4 from Nepal, 3 from Tibet), including 121 individuals that were spatially mapped within a 100 m × 100 m plot. To test for seed flow, the cpDNA *trn*T-*trn*F regions of 100 individuals from 6 populations (4 from Nepal, 2 from Tibet) were also sequenced. Significant genetic differentiation was detected between the two regions by both microsatellite and cpDNA data analyses. These two datasets agree about southern and northern population differentiation, indicating that the Himalayan mountains represent a barrier to *H*. *tibetana* limiting gene flow between these two areas. At a fine scale, spatial autocorrelation analysis suggests significant genetic structure within a distance of less than 45 m, which may be attributed mainly to vegetative reproduction and habitat fragmentation, as well as limited gene flow.

## Introduction

Geographic isolation is considered to play a critical role in the genetic differentiation of plant populations [1], [2]. Physical barriers, such as mountain ranges, rivers, and glaciers may prevent gene flow and cause genetic differentiation of isolated natural populations [1], [2], [3], [4], [5]. Genetic differentiation of plant populations can also result from other biological characteristics or evolutionary processes, such as pollination biogogy [6], mating systems [7], [8], and limited pollen or seed dispersal [9].

**Fig 1:**
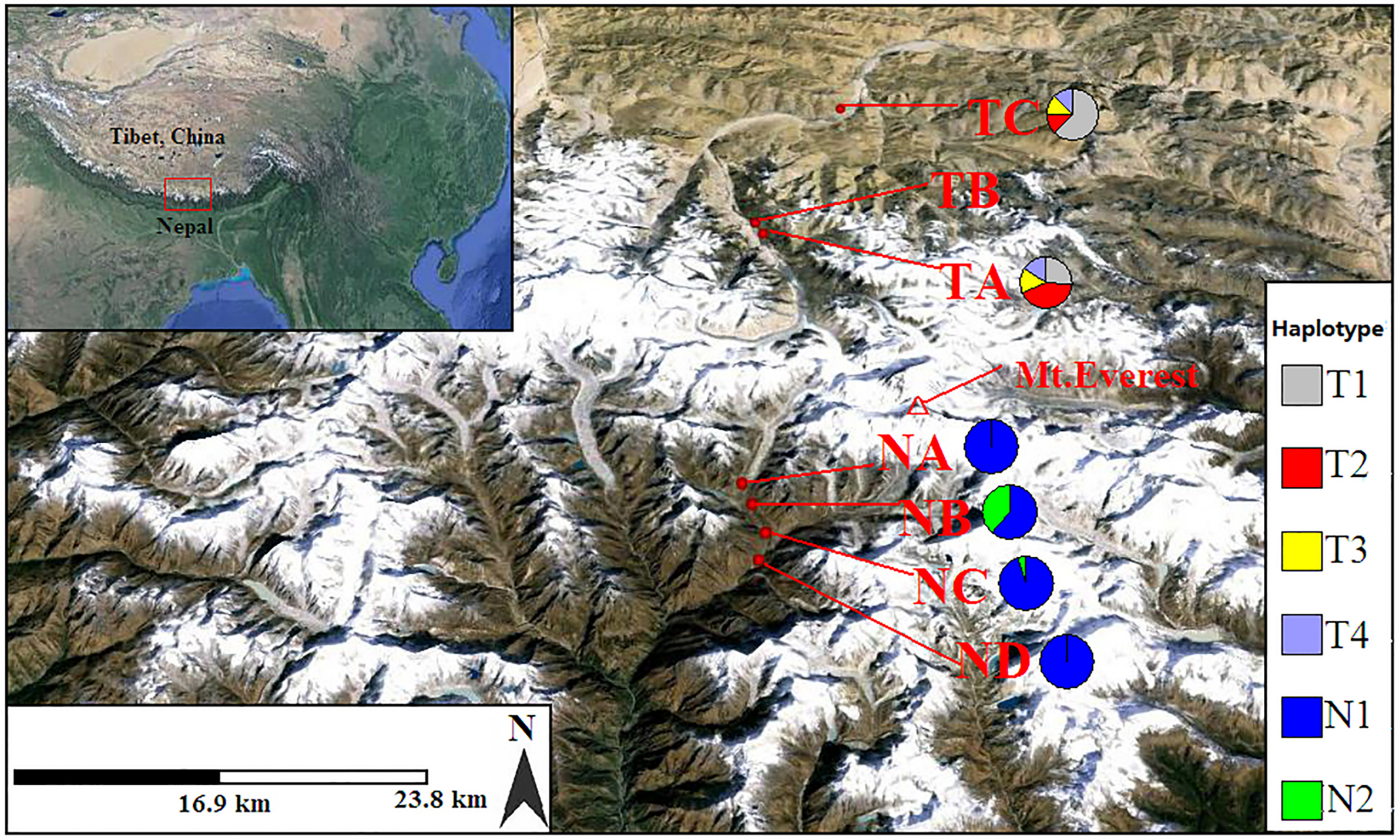
Map of the locations of the seven populations of *Hippophae tibetana* and the distribution of cpDNA haplotypes in four populations (NA, NB, NC, ND) from the south and three populations (TA, TB, TC) from the north of Mt. Everest. For detailed information about the populations, see Table 1. Pie charts show the proportions of haplotypes within each population.

The Himalayan mountain range extends for about 2500 km from west to east [10]. It includes the highest mountains in the world, which are considered to form the biogeographical boundary between the Palaearctic region to the north and the Oriental region to the south [11]. They form a natural barrier between the Tibetan Plateau and the Indian subcontinent [12]. The Himalaya includes Earth’s highest mountain, Mount (Mt.) Everest (8844 m), located on the border between Nepal and Tibet, China (Fig 1). This geographical barrier has resulted in distinct faunal compositions [13] and contrasting vertical vegetation types [14] on the southern and northern slopes of Mt. Everest. As the highest physical biogeographical barrier in the world, Mt. Everest is a perfect area for studying the effect of the Himalayan mountains on genetic differentiation and gene flow of alpine plant populations growing north and south of Mt. Everest. Other studies suggest that the Himalayan range is a very strong dispersal barrier limiting gene flow between southern and northern regions in freshwater snails [11] and for human populations [12]. However, information on how this mountain range influences gene flow and population differentiation in alpine plants between the southern and northern regions is lacking.

Studies on the genetic structure of alpine plants in this region also provide an excellent model for testing intraspecific evolutionary processes and dynamics associated with climate change in this region, because this area is highly sensitive to global climate change [15]. Whether or not cold-adapted alpine species can survive or shift upwards to higher elevations and find optimal new habitats in response to ongoing climate warming [16], [17], [18], [19], largely depends on the ability of alpine plants to disperse from their original habitat and their ability to adapt to new habitats by rapid evolution [16], [20]. Studying the genetic structure of species today can provide informations on their population dynamics in response to past geological or climatic changes and the evolutionary ability of the species [21]. Therefore, it is important to study the gene flow and evolutionary ability of the species today to understand the potential response of alpine plants to future global warming. The study of both fine-scale and macrogeographic genetic structure in alpine plant populations can provide important information about how gene flow, natural selection, and genetic drift collectively influence the genetic variation of plant populations [16], [22].

The major aims of this study are to test the hypothesis at the landscape scale that the Himalayan mountains serve as a physical barrier to gene flow for *H*. *tibetana*, and to explore at a fine scale spatial genetic structure within populations. We use nuclear microsatellite and chloroplast DNA (cpDNA) data to examine intraspecific diversification of *H*. *tibetana*. Microsatellite markers are co-dominant markers that are highly abundant and randomly distributed in genomes and usually highly polymorphic and selectively neutral [23]. For this reason, Microsatellites have been regarded as a powerful tool to analyze gene flow [24], [25], and population genetic structure [26], [27], [28]. cpDNA is maternally inherited in *Hippophae* [29] and the genetic variation inferred from this molecular marker can be very useful to study intraspecific differentiation and species responses to past climate change [30].

The specific objectives of our study are thus: 1) to examine the spatial genetic structure of *H*. *tibetana* at distinct geographic scales in the surroundings of Mt. Everest; and 2) to test the hypothesis that the Himalayan mountains serve as a significant geographical barrier for genetic connectivity between *H*. *tibetana* populations.

## Materials and methods

### Study species

*Hippophae tibetana* Schlect. is a small shrub in the Elaeagnaceae family. It is dioecious and wind pollinated and endemic to the Tibetan-Qinghai Plateau. Its elevational range is 2800 to 5200 m [31] and it is one of the shrubs that occur at very high elevations [30]. On the northern slope of Mt. Everest in Tibet, *H*. *tibetana* has an elevational range from 4200 m to 5200 m, while it occurs from 4060 m to 4620 m on the southern slope in Nepal. It is easy to identify and distinguishable from other species within the genus by its verticillate leaves of three and short (7~80 cm) height [32]. It mainly occurs along alpine river banks, on sandy flood plains and valley terraces, and in grassland. It is the sister taxa of all extant species of the genus *Hippophae* [33].

### Ethics statement

Special permissions for collecting leaf samples were not required because *H*. *tibetana* is not an endangered or protected species. Field work and sampling were carried out in the southern and northern valley of Mt. Everest and Mountain Qomolangma National Nature Preserve (QNNP) and all the required permits for sampling in this protected area were obtained. Geographic coordinates for the seven sampling locations are given in Table 1.

**Table 1:** List of populations analyzed in the present study with their sampling localities, number of specimens, coordinates, genetic diversity parameters, and cpDNA haplotype composition of each population and their frequencies.

Population	*N*	Elevation	Latitude	Longitude	Nuclear microsatellites				Chloroplast haplotype			
		(m)	(N)	(E)	*Fis*	*H*_o_	*H*_e_	*P*	*N*	Haplotypes (Frequencies, %)	*D*	*π*
NA (Nepal)	20	4614	27°55′23″	86°48′48″	0.342	0.238	0.306	87.50	16	N1 (100)	0.0000	0.00000
NB (Nepal)	20	4439	27°54′56″	86°48′22″	-0.183	0.300	0.256*	50.00	19	N1(61), N2(39)	0.5033	0.00059
NC (Nepal)	20	4230	27°52′99″	86°49′22″	-0.175	0.263	0.215	62.50	20	N1 (95), N2(5)	0.1000	0.00011
ND (Nepal)	20	4069	27°51′98″	86°48′91″	-0.167	0.219	0.175	62.50	18	N1(100)	0.0000	0.00000
TA (Tibet)	20	5041	28°09′43″	86°50′36″	0.422	0.106	0.216	62.50	14	T1(26), T2(42), T3(16), T4(16)	0.7526	0.00193
TB (Tibet)	20	5030	28°10′04″	86°50′24″	0.073	0.131	0.130	50.00				
TC (Tibet)	121	4410	28°18′48″	86°55′37″	0.275	0.211	0.306*	100.0	13	T1(61), T2(13), T3(13), T4(13)	0.5238	0.00166
Mean TC-sub dataset	20				0.084 0.313	0.209 0.202	0.229 0.271	67.85 72.50				

*N*, number of individuals analyzed; *F*is, Inbreeding coefficient at population level; *H*_o_, observed heterozygosity; *H*_e_, expected heterozygosity;

*, significant Hardy-Weinberg disequilibrium; *P*, the percentage of polymorphic loci; *D*, estimates of gene diversity; *π*, nucleotide diversity averaged across loci; TC-sub dataset rows show the results obtained for TC population after resampling performed to control the effect of sample size.

### Sampling sites and sampling strategy

Mt. Everest is located in the Mahalangur section of the Himalaya (27.98°N, 86.92°E). It lies on the border between Tibet and Nepal, which runs across the summit point.

In order to investigate the role of the Himalayan mountains as a geographical barrier to gene flow, a total of seven *H*. *tibetana* populations comprising four Nepalese populations (south slope of Mt. Everest) and three Tibetan populations (north slope of Mt. Everest) were collected in June and August 2013 (Fig 1, Table 1). For each population, 20 individuals were randomly selected except for population TC (Tibetan population C) where 121 individuals were collected for fine-scale genetic structure analyses. Those populations from the same region, such as Nepalese populations NA, NB, NC, and ND, as well as Tibetan populations TA and TB are geographically close to each other, but belong to distinct elevational ranges and patches. Although we are not sure whether these different patches of *H*. *tibetana* from the same region or same valley belong to one or more populations, we treated different patches at different elevations as different populations in this study.

**Fig 2:**
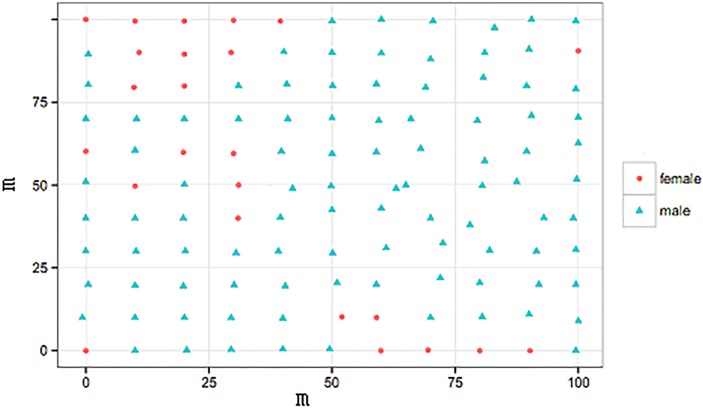
Spatial distribution of the 121 individuals within a plot (population TC, 100 m x 100 m) sampled from the north slope of Mt. Everest.

In addition, to increase the geographic resolution of genetic analyses and infer dispersal distance in *Hippophae tibetana*, we established a 100×100 m plot (Fig 2) within population TC (located on the northern slope of Mt Everest in the Rongbuk valley, Tibet; see Fig 1, Table 1). Since *H*. *tibetana* is a clonal plant and has a high density at the sampling site (and it is not possible to map all the individuals within the plot), we mapped the position of all *H*. *tibetana* individuals that were at least 5~10 m apart from each other. A total of 121 individuals were mapped. Young and fresh leaves for DNA analysis were collected from 7 populations and immediately placed in silica gel in the field.

The chloroplast *trn*T-*trn*F sequences of 100 individuals of *H*. *tibetana* from 6 populations from the northern and southern slopes of Mt. Everest (4 from Nepal, 2 from Tibet) (Fig 1,Table 1) were used for testing the role of Himalayan mountains as a seed dispersal barrier by comparing them with all B-lineage chlorotypes of Tibetan populations from our previous study [30]. The sampled individuals in each population were all more than 10 m apart.

### DNA extraction and PCR amplification and sequencing

Total genomic DNA was extracted from the silica gel-dried leaves of *H*. *tibetana*, using the Plant Genomic DNA Kit (Tiangen Biotech, Beijing, China).

We used 25 individuals from different Tibetan populations to develop microsatellites and originally developed 16 nuclear microsatellite loci for seven populations using 5´-anchored PCR [34], eight (Table 2) of which gave reproducible and clear banding patterns, and exhibited popymorphism among individuals from the seven populations. All the primers had reliable scoring and interpretation of the electropherograms was performed by the same person in the same laboratory for all samples. Amplification reactions for the extracted DNA samples were carried out on a total volume of 10 μL containing approximately 10 ng of genomic DNA, 5μL 2×Taq PCR MasterMix (Tiangen Biotech), 0.4 μM of M13-tailed forward primer, 0.4 μM of reverse primers, and 0.36 μM of M13 primer labeled with either 6-FAM or ROX fluorescent dyes. PCRs were performed by an initial denaturation at 94°C for 3 min; followed by 33 cycles of denaturation at 94°C for 30 s, annealing at the specific annealing temperature for 30 s, and 72°C for 30 s, plus a final extension of 72°C for 7 min. Different fluorescently labeled PCR products were mixed and then analyzed on an ABI 3710XL DNA analyzer (Applied Biosystems) using LIZ-500 as the internal size standard (Applied Biosystems). All these procedures follow Zeng et al.[35] (2012).

**Table 2:** Characteristics of 8 polymorphic microsatellite markers developed for *Hippophae tibetana*.

Locus	Genebank Accession	Primer sequence (5´-3´)	Repeat motif	Ta (°C)	Size range (bp)	NA	*H*_e_	*H*_o_	*Nm*	*Fis*
HS1	JF268791	AACCACAGCAAAACAAAAAAC	(TGA)8	53	241–253	3	0.131	0.107	0.096	0.774
		TAAAAATACACCTCCAACTCA								
HS2	EU429318	CCATCCACATTCCTCTTCAA	(GAATGT)3	57	135–146	8	0.164*	0.096	0.113	0.688
		GTCATTACCCACCTTCACAT								
HS3	EU429317	CCCCCTTCTTTTTCAGATAGT	(A)10	57	136–148	3	0.008	0.008	13.298	0.018
		GAGAGTTGCATTTTTGCCCTTT								
HS4	EU429310	CAATTGTTCAATACTAAATG	(A)6(CAAACA)3	57	118–148	6	0.545	0.655	1.484	0.144
		ATCCTAATCAAAAGAAATC								
HS5	EU429312	TGCCAGAAGATTAGACTTTTAC	(A)8(GAA)4	55	85–91	3	0.245	0.375	0.944	0.209
		GGAGCAGCTTATACCCATTAC								
HTP-18	EU429314	ACGGGAGAAAAAGAATGAATAA	GA)6…(GA)8	57	123–127	3	0.251	0.247	0.190	0.569
		TCTTCTGTCTCTTGCTTACT								
HTP-21	EU429315	GACGCTTGGCGACAATATAACA	A)8…(T)6	57	146–148	6	0.292	0.126	0.446	0.359
		CAAACCCATAGCCTCTACCTCC								
HTP-26	EU429316	AGAGAGAGACTGATTGA	(TGTA)3	57	241–248	3	0.195*	0.064	0.155	0.617
		AAAATAATAGCGTGGGAGAA								
Mean						4.375	0.288	0.209	2.09	0.422

Ta, annealing temperature; NA, number of alleles; *H*_o_, observed heterozygosity; *H*_e_, expected heterozygosity,

*, significant Hardy-Weinberg disequilibrium;

*N*_*m*_, gene flow; *F*is, Inbreeding coefficient at microsatellite loci level.

We amplified the *trn*T-*trn*F region of cpDNA of 100 individuals from the 6 populations using the primers ‘a’ and ‘d’, and ‘c’ and ‘f’ [36], following Wang et al. (2010) [30].

### Data analyses for nuclear microsatellite data

To characterize the genetic diversity within populations and among populations for each nuclear microsatellite locus and each population, various genetic parameters such as the percentage of polymorphic loci (*P*), number of different alleles (*NA*), observed heterozygosity (*Ho*), expected heterozygosity (*He*), and gene flow (*Nm*) were calculated using genALEX 6 software [37]. Test of deviation from the Hardy-Weinberg equilibrium (with a 0.01 significant) among microsatellite markers and populations were performed using PopGene. Since the population TC had 121 individuals and other the six populations each had 20 individuals, a resampling procedure was performed to control for the confounding effect of disproportional sample size. We repeated a resampling procedure 10 times and the means of estimations for each genetic parameter based on the resampled dataset were calculated.

To investigate the spatial genetic structure of the seven populations of *H*. *tibetana* based on nuclear microsatellite data, the genetic differentiation among populations was estimated by calculating *F*st. Four methods were used for analyzing genetic structure. First, the effect of distance in population differentiation was analyzed by a Mantel (1967) [38] test with 1000 random permutations to estimate and evaluate the correlation between geographic distances and genetic distances measured by *F*st [39]. Second, to detect the geographic structure of genetic variation, and especially to test the effect of the Himalayan mountains on the genetic structure of *H*. *tibetana* populations on its two sides (the two sides were regarded as two regions), an analysis of molecular variance (AMOVA) was performed, in which the total genetic variance can be seen at different levels, including among different regions, among populations, and within populations. Third, to examine the spatial genetic pattern of all 241 individuals visually, a principal component analysis (PCA) was performed and the results presented in a two-dimensional plot. All these analyses were conducted using genALEX 6. Finally, STRUCTURE 2.1 [40] was used to assess the pattern of genetic variation among populations. A Bayesian clustering analysis was performed to group the 241 individuals into different groups based on their allelic frequencies and multi-locus genotypes. STRUCTURE was run for *K* = 2 to *K* = 8 clusters to examine the gene exchanges among the seven populations and between the two regions, respectively. Since the admixture model allowed for analyzing admixture and correlated allele frequencies, the admixture model was used. We ran five independent simulations, each with a burn-in of 100,000 generations and a subsequent Markov chain of 1,000,000 generations. We calculated the final posterior probability of *K*, ln*p* (x|*K*) by using the analyses with the highest probability for each *K*. ln*p* (x|*K*) usually levels or increases slightly after the ‘ appropriate *K*’ is reached [41]. We calculated Δ*K* following Evanno et al. (2005) [42].

To investigate the particular spatial genetic extent of *H*. *tibetana* within population TC, we examined the pattern of fine-scale genetic structure based on nuclear microsatellite data by autocorrelation analysis with a heterogeneity test [43] using genALEX 6.5 [44], and 10 lags were considered. A randomization test with 1,000 permutations was also applied to assess for significant deviations from a random spatial distribution.

### Data analyses of the chloroplast data

The cpDNA sequences of 100 individuals of 6 populations (Table 1) were aligned using the CLUSTAL X program [45] with additional minor manual adjustments, and assigned to different haplotypes using DnaSP 5.10 [46]. We calculated the indices of gene diversity (*D*) and nucleotide diversity (π) [47] for each population using ARLEQUIN [48].

In order to examine the phylogenetic relationships of haplotypes, the chlorotypes obtained in the present study and all the B-lineage chlorotypes from our previous study [30] (all from the northern side of the Himalayan mountains and western Tibetan area) were used. Phylogenetic analyses were carried out by neighbor joining (NJ) and maximum parsimony (MP) using MEGA version 4 [49], as well as by maximum likelihood (ML) using PhyML (http://www.atgc-montpellier.fr/phyml/). Gaps were regarded as missing in all analyses. In the ML analysis, we used MODELTEST [50] to select parameters and assumptions and chose the general-time reversible (GTR) model [51]. In the MP analyses, we carried out a heuristic search with 100 random-taxon addition replicates with TBR branch swapping. We calculated bootstrap values to assess branch support using 1000 replicates. All the A-lineage chlorotypes from our previous study [30] were also chosen as an out group and divergence time in the haplotype tree was estimated according to the our previous study [30]. To define groups of six populations of *H*. *tibetana*, we performed spatial analysis of molecular variance using SAMOVA 1.0 [52] (http://web.unife.it/progetti/genetica/Isabelle/samovar.html) and two groups were assumed. A haplotype network was constructed using NETWORK version 4.5.1.0 (http://www.fluxus-engineering.com) using the median joining (MJ) method [53] and MP calculations [54]. Two parameters of genetic diversity (*H*S and *H*T) and two of differentiation (*G*_ST_ and *N*_ST_) were estimated using PERMUT (http://www.pierroton.inra.fr/genetics/labo/Software/Permut/). We also compared the two estimates of population divergence *G*_ST_ (coefficient of genetic variation over all populations) [55] and *N*_ST_ (coefficient of genetic variation affected by both haplotype frequencies and genetic distances between haplotypes) by using PERMUT with a permutation test with 1000 permutations.

## Results

### Genetic diversity within populations

We used eight microsatellite loci to characterize the genetic diversity in seven populations of *H*. *tibetana*. Genebank accession numbers of the eight microsatellite loci are HS1—JF268791, HS2—EU429318, HS3—EU429317, HS4—EU429310, HTP18—EU429314, HTP21—EU429315 and HTP26—EU429316 (Table 2). All these eight primer pairs gave clear and polymorphic peaks with a maximum of two alleles for each locus per individual. Estimates of the diversity among the nuclear microsatellite loci are shown in Table 2. The smallest allele is 85 bp for S5 and the largest is 253 bp for locus S1. In total, there are 34 alleles for seven populations. Two of primers (S2, HTP-26) and two of populations (NB, TC) exhibit a significant deviation from the Hardy-Weinberg equilibrium (Tables 1 and 2). After the confounding effect of disproportionate sample sizes was controlled for by resampling, the highest proportion of polymorphic loci and genetic diversity is shown by population NA (*P* = 87.5, *H*e = 0.306) (Table 1), while the lowest is presented by population TB (*P* = 50, *H*e = 0.130). The mean values for the observed heterozygosity (*H*o) and the expected heterozygosity (*H*e) in seven populations are 0.209 and 0.288, respectively (Table 2). Gene diversity (*D*) within the six populations ranges from 0 to 0.75 (Table 1), and nucleotide diversity (π) ranges from 0 to 0.0019 based on the chloroplast data. The mean gene diversity within population (*H*_S_) is 0.331 and total gene diversity (*H*_T_) is 0.668 (Table 3).

**Table 3:** Estimates of the average gene diversity within population (*H*s), total gene diversity (*H*T), interpopulation differentiation (*Gs*t), and number of substitution types (*N*st) of the *Hippophae tibetana* populations based on cpDNA data.

No. of populations	No. of alleles	*H*_S_	*H*_T_	*G*st	*N*st
6	6	0.331	0.668	0.504	0.570

### Spatial genetic structure among populations at the landscape scale

**Fig 3:**
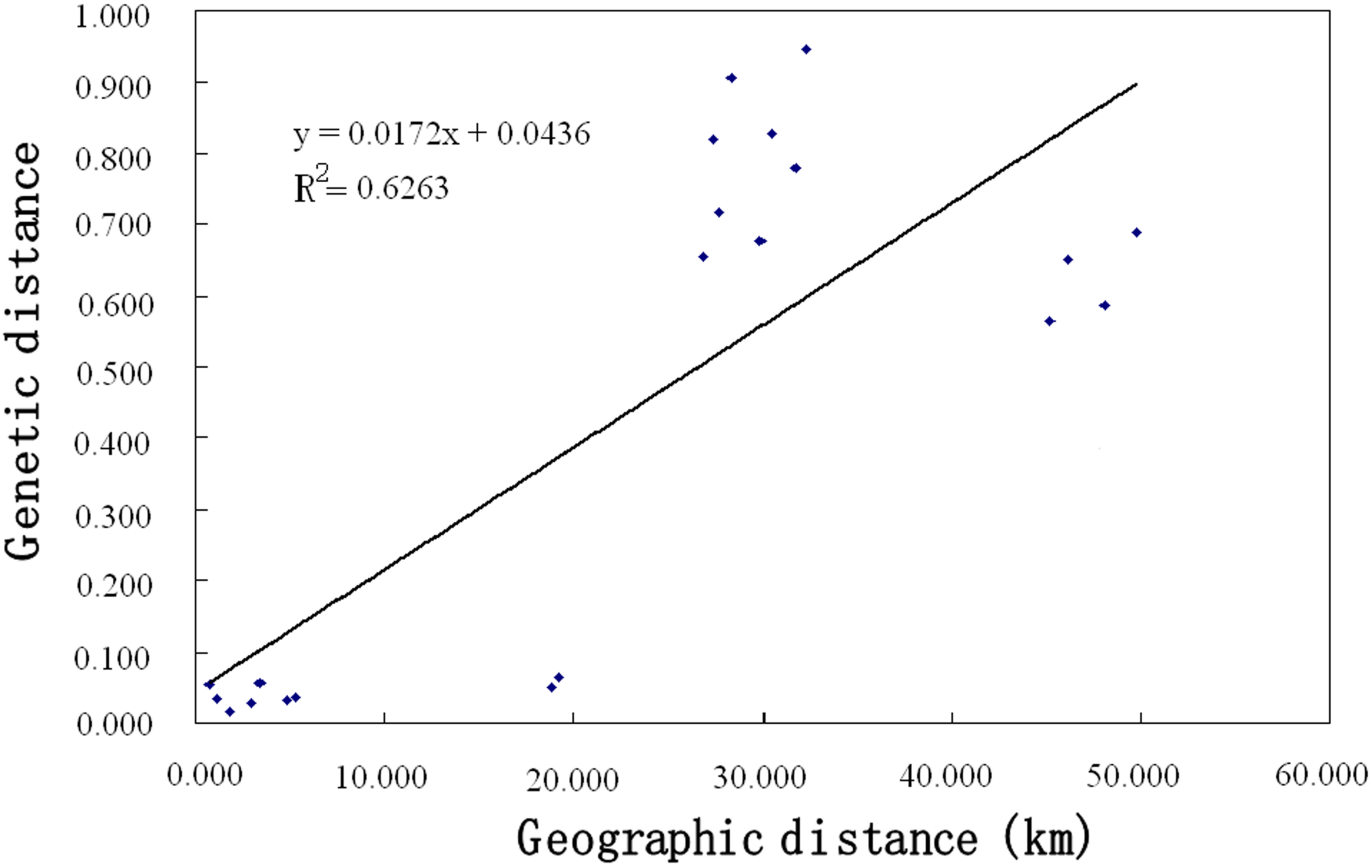
Relationship between genetic and geographic distance in *Hippophae tibetana* based on the Mantel test.

**Fig 4:**
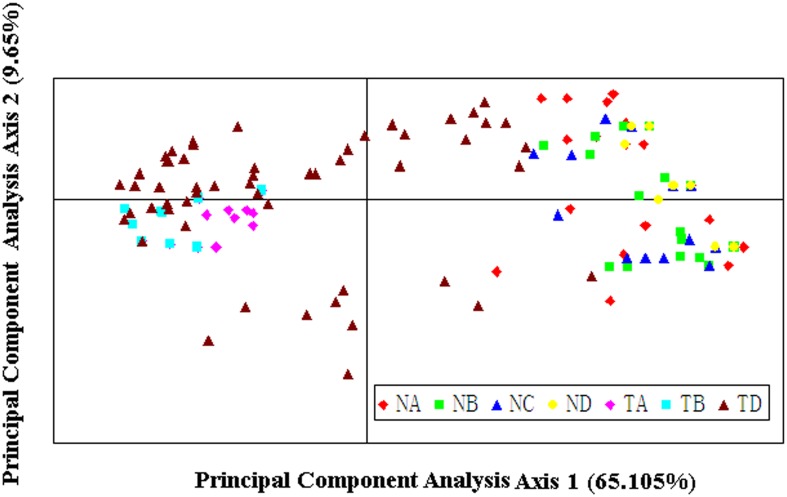
PCA results showing 241 individuals of *Hippophae tibetana* from seven populations based on nuclear microsatellite multi-locus genotypes. The different colors represent the different populations from both slopes of Mt. Everest (NA, NB, NC, and ND from the southern slope, TA, TB, and TC from the northern slope).

**Fig 5:**
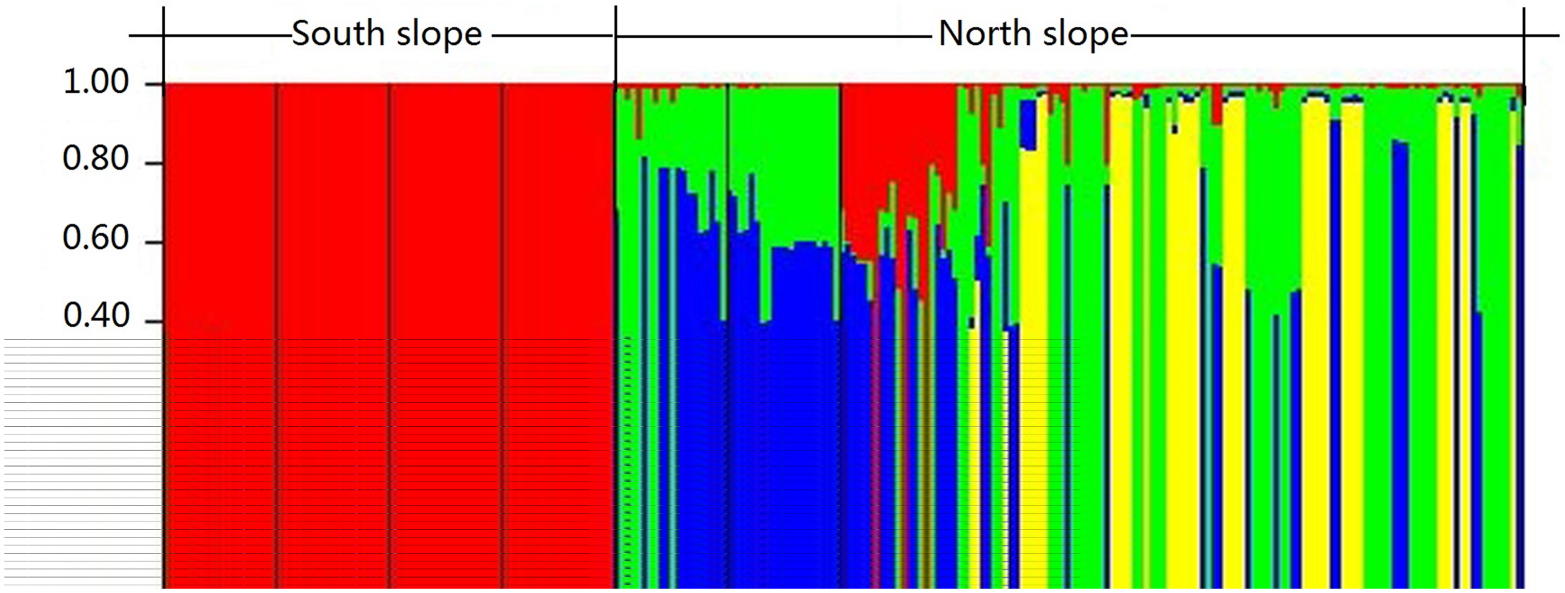
Bayesian inference of the number of clusters (*K* = 4) of *Hippophae tibetana*. *K* was estimated using the posterior probability of the data given each *K* and the distribution of Δ*K*. The four colored clusters were detected from the STRUCTURE analysis. For detailed information about populations NA, NB, NC, ND, TA, TB, and TC, see Table 1.

A very high level of genetic differentiation is detected among the seven populations with *F*st = 0.439 (Table 4). The results of AMOVA based on nuclear microsatellite data show that most of the total genetic variation (64%) and the highest estimated variation (5.264) are found *among regions*, and the effect of region on genetic variation is statistically significant (*P* = 0.01) (Table 4), indicating that there is high variation in *H*. *tibetana* populations between the two slopes of Mt. Everest. Intermediate genetic variation (31%) and estimated variation (2.535) are *within populations*, whereas the lowest genetic variation (6%) and estimated variation (0.475) are found *among populations* (Table 4). The Mantel test detects a significant positive correlation between genetic distance and geographical distance (*R*^*2*^ = 0.6263, *P* = 0.01) (Fig 3) for the seven populations. In other words, there is a significantly positive correlation between genetic and geographic distances. Principal component analysis (PCA) provides further insights: the first two PCA axes explain 65.1% and 9.65% of the total genetic variation, respectively (Fig 4). The two-dimensional PCA plot highlights the differentiation between the two regions (Fig 4), further suggesting the Himalayan mountains as a barrier to gene exchange. We estimated the number of natural genetic groups to be *K* = 4 according to the calculation of Δ*K*. The population cluster results of STRUCTURE further indicate that there is only a little gene exchange between the two regions (Fig 5), and reveal a clear geographical boundary between the northern- and southern-region populations.

**Table 4:** Analysis of molecular variance (AMOVA) based on nuclear microsatellites data for seven populations and chloroplast (cp DNA) data for six populations of *Hippophae tibetana* in the Mt. Everest area.

Nuclear microsatellites								Chloroplast DNA				
	df	Est. Var	Percentage variation	Ф	*P* value	*F*is	*F*st	df	SS	VC	PV	Fixation indices
Whole-dataset						0.422	0.439					
Among regions	1	5.264	64%	0.636	0.01			1	56.367	1.389	69.71%	F_SC_ = 0.113*
Among populations	5	0.475	6%	0.158	0.01			4	6.642	0.068	3.43%	F_ST_ = 0.731*
Within population	234	2.535	31%	0.694	0.01			94	50.271	0.534	26.86%	F_CT_ = 0.697*
Nm	0.199											
**Sub-dataset**						0.127	0.440	Total	113.280	1.990	100%	
Among regions	1	5.878	69%	0.698	0.01							
Among populations	5	0.491	7%	0.195	0.01							
Within populations	133	2.030	25%	0.757	0.01							
Among regions	1	5.878	69%	0.698	0.01							
*N*_m_	0.102											

The values of whole dataset show the results of original dataset, while the values of sub-dataset show the results with the confounding effect of disproportionate sample size controlled by resampling procedure; df, degree of freedom; Est.Var, estimated variance; Ф, Ф-statistics; *F*is, Mean inbreeding coefficient at population level; *F*st, Mean population differentiation; *Nm*, Mean gene flow; VC, Variance of components; PV, percentage of variation; F_SC_, differentiation between two groups; F_ST_, differentiation among populations; F_CT_, differentiation within populations.

**Fig 6:**
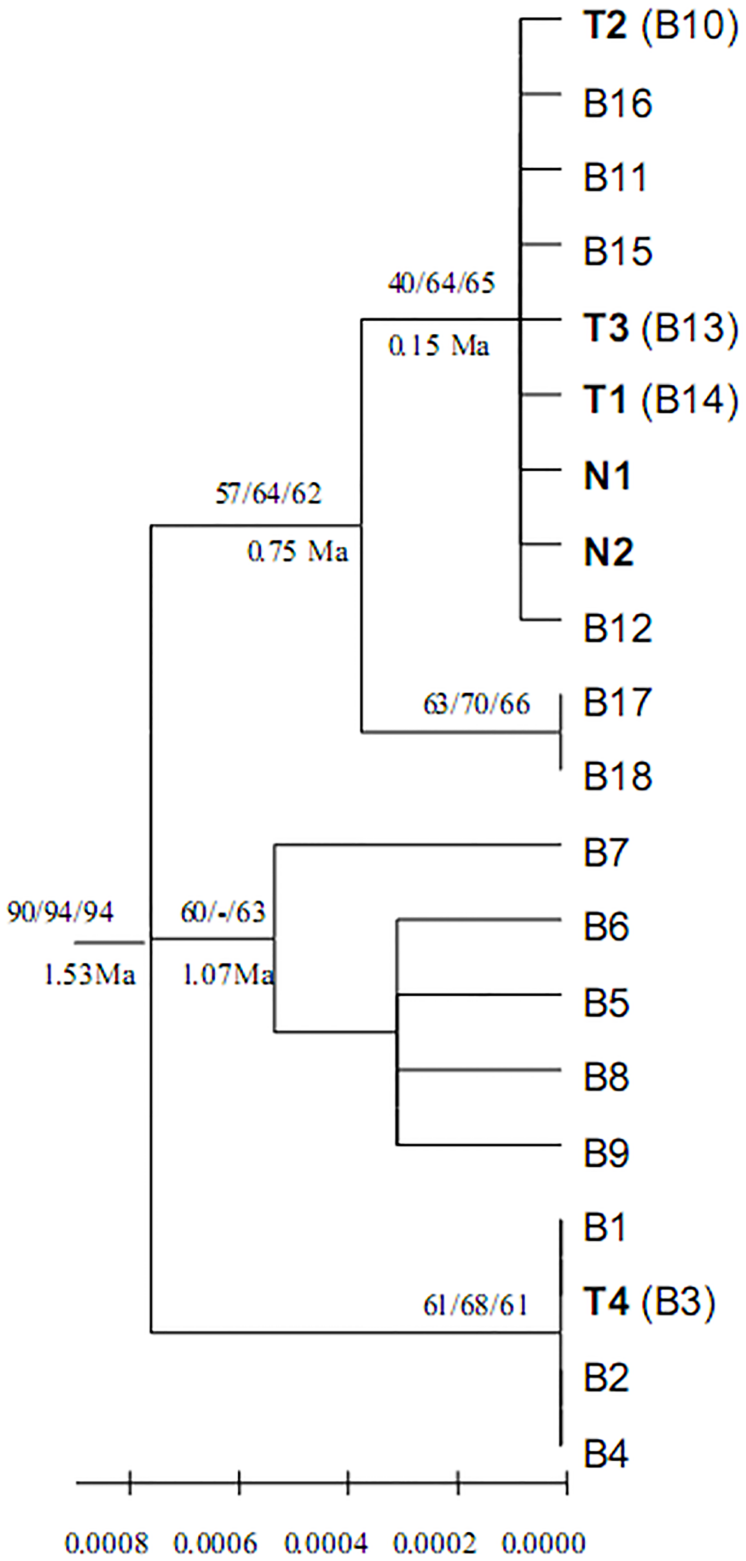
The neighbor-joining tree topology of the 6 cpDNA chlorotypes detected from the *trn*T-*trn*F region of *Hippophae tibetana* in this study, which are indicated by bold letters. T1, T2, T3, and T4 are B14, B10, B13, and B3 in our previous study (Wang et al., 2010), respectively, while N1 and N2 are new chlorotypes first found on the southern slope of Mt. Everest. The other B-lineage chlorotypes were found on the north slope of the species distribution range of the northern Himalaya in our previous study (Wang et al. 2010). Numbers below the branches indicate the bootstrap values for neighbor-joining (left number above the branches), maximum parsimony analyses (middle number above the branches), and maximum likelihood (right number above the branches) analyses, respectively; numbers below the branches indicate inferred dates in Ma before present.

The number of groups (*K*) is set to two in the spatial genetic analysis of haplotypes using SAMOVA. Two groups are identified, which are populations NA, NB, NC, and ND from the southern slope and populations TA and TC from the northern slope. SAMOVA suggests a barrier that separates the Nepalese populations from Tibetan populations. However, this grouping is not consistent with the phylogentic analysis, because haplotypes T1, T2, and T3 from northern populations and haplotypes N1 and N2 from southern populations are grouped together into the one clade (Fig 6). AMOVA of the cpDNA data suggests that approximately 69.71% of the total genetic variation is assigned to between two groups or two regions (Table 4), i.e. southern and northern populations; while about 26.86% of the total genetic variation is within populations, and only 3.43% of the total genetic variation occurs among populations. The F_SC_, F_ST_, and F_CT_ values are 0.113, 0.731, and 0.697, respectively, and are statistically significant (Table 4). Population differentiation across Mt. Everest is high; the two coefficients of genetic differentiation (*G*_ST_ and *N*_ST_) over all populations are 0.504 and 0.570 (Table 3), respectively. The test for phylogeographic structure of haplotype variation across the distribution of the species shows that *N*ST is larger than *G*ST, but not significant (*N*ST > *G*ST, *P* > 0.05).

### Spatial genetic structure within populations at a fine scale

**Fig 7:**
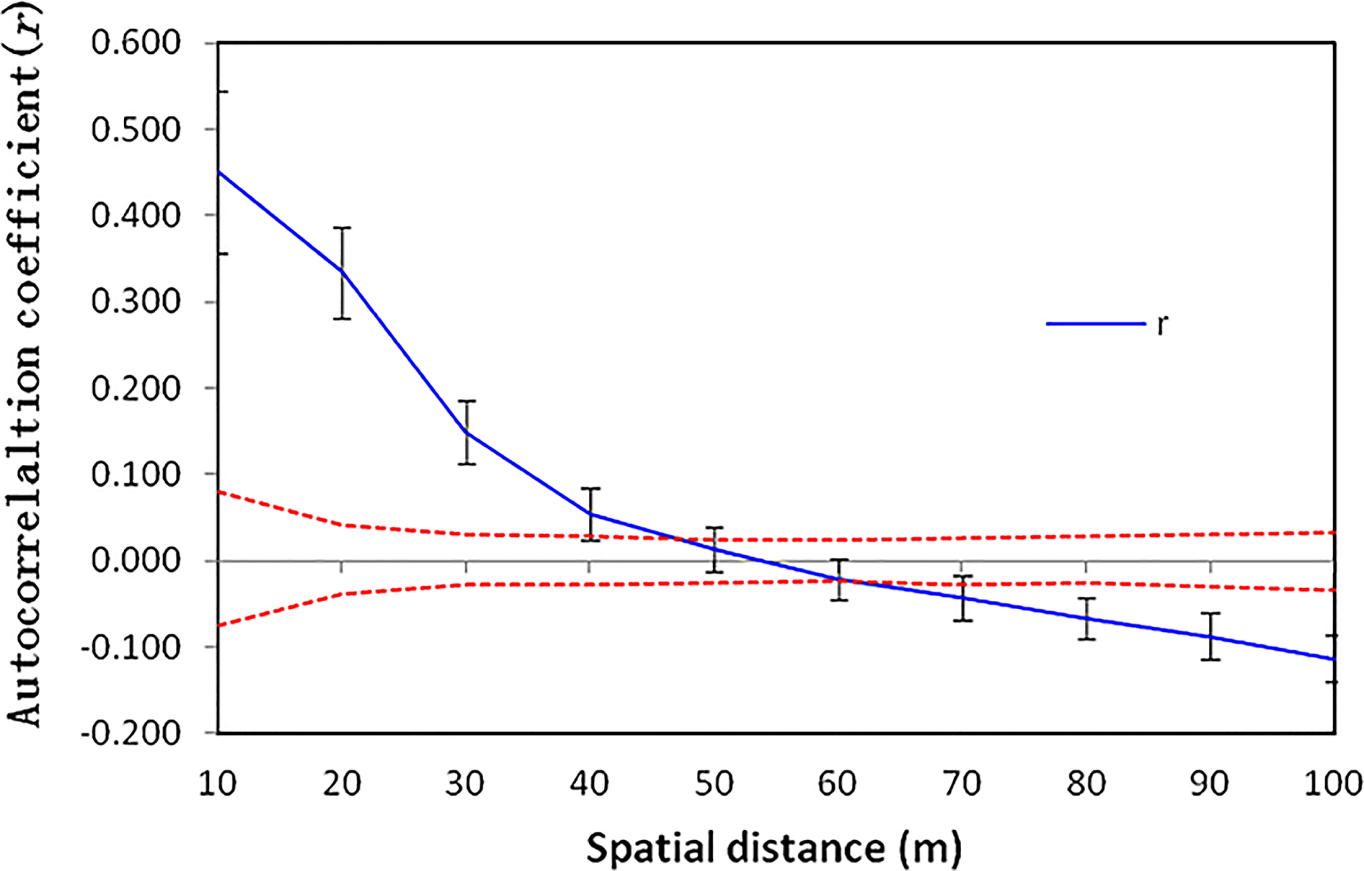
Correlogram of autocorrelation coefficients for 121 individuals of *Hippophae tibetana* within a 100 m x 100 m plot (population TC). Dashed line represents the upper and lower 99% confidence limits around zero autocorrelation.

Based on the whole dataset of nuclear microsatellites for the TC population consisting of 121 individuals, fine-scale spatial genetic structure was examined. Correlograms calculated at various distance classes with 99% confidence intervals are given in Fig 7. There is a statistically significant spatial positive autocorrelation among individuals located up to 45 m apart approximately, while at distances larger than 60 m apart the autocorrelation becomes statistically significantly but negative (Fig 7).

### Phylogenetic relationships and haplotype distribution of *Hippophae tibetana*

Three different phylogenetic analyses, NJ (neighbor joining), MP (maximum parsimony), and ML (maximum likelihood) produce a similar topology (Fig 6). Two main clades are identified among the six haplotypes in the present study. Genebank accession numbers of the six haplotyes are T1—KP208944, T2—KP208945, T3—KP208946, T4—KP208947, N1—KP208948 and N2—KP208949. Although southern and northern populations have completely different haplotypes, their relationships between the clades are not well resolved (Fig 6). One clade contains five haplotypes (T1, T2, T3, N1, N2) with bootstrap values of 40%, 64%, and 65% for neighbor-joining (left number above the branches) (Fig 6), maximum parsimony analyses (middle number above the branches), and maximum likelihood (right number above the branches) analyses, respectively, in which haplotypes N1 and N2 are endemic to the southern region of Mt. Everest, whereas haplotypes T1, T2, and T3 are from the northern region. The remaining northern haplotype T4 formed an independent clade together with other B-lineage haplotypes B1, B2, and B4 (Fig 6).

**Fig 8:**
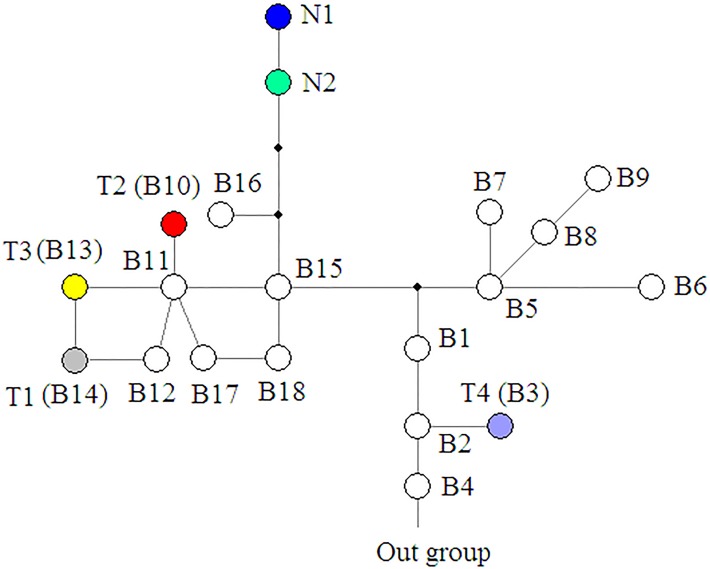
Chlorotype network based on *trn*T-*trn*F sequences of *H*. *tibetana*. Colored circles indicate 6 chlorotypes found in the present study. T1, T2, T3, and T4 are B14, B10, B13, and B3 in our previous study (Wang *et al*., 2010), respectively, while N1 and N2 are new chlorotypes first found on the southern slope of Mt. Everest in Nepalese populations. The other circles represent the remaining B-lineage chlorotypes from our previous study (Wang *et al*., 2010). The network was constructed using NETWORK version 4.5.1.0 (http://www.fluxus-engineering.com) with median joining and maximum parsimony methods. Black dots represent missing chlorotypes.

A network of the six haplotypes found in this study and other haplotypes of B-lineage from the study of Wang et al. (2010) [30] shows that the six haplotypes found in the present study are almost scattered in the B lineage network (Fig 8). However, haplotypes N1 and N2 from the southern region are close to each other and occur at the end of one clade with two mutation steps, and these two haplotypes are connected to haplotype B16 that is distributed northwest of Mt. Everest. Except for haplotype T4, the remaining haplotypes, T1, T2, and T3 are also close to each other in the network.

## Discussion

### Within-population genetic variation in *Hippophae tibetana*

In general, wind-pollinated, outcrossing woody species are supposed to have high genetic diversity within populations [29], [56]. Based on nuclear microsatellite data analyses, our estimates for the dioecious, wind-pollinated shrub *H*. *tibetana* show relatively high levels of within-population genetic variation (*H*e = 0.288: Table 2) compared to other species in the same genus. This is larger than the values obtained from three *H*. *rhamnoides* subspecies using ISSR markers, such as *H*. *rhamnoides* ssp. *yunnanensis* (*H*e = 0.199), ssp. *sinensis* (*H*e = 0.216), and ssp. *gyantsensis* (*H*e = 0.137) [57]. This relatively high diversity within the genus corresponds well with the results of Sheng et al. (2006) [58] who find that the highest diversity among species in the genus of *Hippophae* based on RAPD markers is in both *H*. *tibetana* and *H*. *goniocarpa*. However, compared to other woody, dioecious, wind-pollinated species, Nei’s genetic diversity in *H*. *tibetana* may be regarded as somewhat lower than expected, compared with, for example, *Populus tremuloides* and *P*. *grandidentata*, whose values are 0.30 and 0.35 based on RPAD markers [59], respectively. This may be attributed to the high degree of vegetative reproduction and spatial isolation of natural populations of *H*. *tibetana*, because vegetative reproduction and highly isolated natural populations generally have low genetic diversity within populations [60], [61]. Our field observations also indicate that it is common for *H*. *tibetana* to perpetuate itself clonally by rhizomes (determined by our excavation of plants in the field), and that the natural habitats of *H*. *tibetana* are strongly isolated in our study area.

Both of the average *F*is values of populations (*F*is = 0.084) (Table 1) and microsatellite markers (*F*is = 0.422) (Table 2) are positive, respectively, indicating deficiencies of heterozygotes within populations [62]. In general, population structure and selfing could result in heterozygote deficiency [63]. As *H*. *tibetana* is a dioecious species, its vegetative reproduction or inbreeding might account for heterozygote deficiencies [64].

After the confounding effect of disproportionate sample sizes is controlled for by resampling, the southern population with the highest genetic diversity of microsatellite is in the NA population at the highest elevation (*He* = 0.306, elevation = 4614 m) (Table 1), while the northern population with the highest genetic diversity is in the TC population at the lowest elevation (*He* = 0.271, elevation = 4410 m) (Table 1). These results seem to contradict the general pattern that populations at intermediate elevations have greater genetic diversity than populations at lower or higher elevations [65]. The cpDNA data analyses which show that estimates of gene diversity (*D*) and nucleotide diversity (*π*) in populations NA and ND are 0 (Table 1). These different results suggest that the different molecular marker used could affect the measure of diversity for a given genome of species. It is also likely that a selective sweep on cpDNA and different mutation rates of microsatellite on the two regions affect the genetic diversity index of species.

### Spatial autocorrelation analysis of *Hippophae tibetana*

The spatial genetic structure within populations of many clonal plants is the result of the joint effect of pollen and seed dispersal [66]. The most likely cause of spatial genetic structure is the formation of local pedigree structure as a result of limited gene dispersal [67]. Analysis of fine-scale genetic structure in population TC based on nuclear microsatellite data reveals a statistically significant positive spatial autocorrelation among individuals of *H*. *tibetana* located up to 45 m apart (Fig 7), suggesting that individuals of *H*. *tibetana* within a distance of less than 45 m are genetically similar to each other, whereas when the distance is larger than 60 m, the genetic similarity to each other decreases. In other words, the genetic patch-width within a population is at least 45 m. The pattern of positive autocorrelation at short distances and negative autocorrelation at larger distances for *H*. *tibetana* may result from two main causes. First, clonal propagation is thought to be one possibility for the spatial genetic structure [68]. As mentioned above, we observed that in the field, fragments of the same clone often appeared as different clones of *H*. *tibetana*, when in fact their below-ground roots were connected. However, this is somewhat contradictory to the estimates of high genetic diversity (*He* = 0.306) found within population TC (Table 1), because if its 121 individuals reproduce largely by vegetative means, it may seem surprising that high genetic diversity is detected within this population. It suggests that both clonal and seed reproduction may coexist in reproductive systems within this population, and it is not necessarily the case that clonal reproduction is always correlated with a reduced level of reproduction by seeds [69]. Fine-scale spatial genetic structure and reproduction by seeds at high elevations in alpine habitats have also been found for *Androsace tapeta* [16]. Second, the fine-spatial genetic structure has also usually been interpreted as a consequence of an isolation-by-distance process with restricted pollen dispersal and seed dispersal within plant populations [68], [70], [71], [72]. In our study, direct quantitative data on pollen and seed dispersal in *H*. *tibetana* are not available, but previous research on congeneric species, such as *H*. *rhamnoides* ssp. *sinensis* shows that the distance of its pollen dispersal is less than 12 m [73]. *H*. *tibetana* may have a similar pollen-dispersal distance. For this reason, we speculate that the relatedness among neighboring individuals is due to localized pollen and seed dispersal, as well as vegetative reproduction of *H*. *tibetana*.

### High population differentiation between two regions and the effects of the Himalayan mountains on population differentiation in *Hippophae tibetana*

When we compare population differentiation, as estimated from nuclear microsatellite loci, with the differentiation-based cpDNA data analyses, the results approximately agree. Both nuclear microsatellite and cpDNA data analyses indicate high population differentiations of *H*. *tibetana* between the two regions. For the nuclear microsatellite data, significant population differentiation is indicated by high *F*st values (*F*st = 0.439, Table 4), and AMOVA indicates that 64% of the total genetic variation is attributable to regional differences and 31% of the total variation is within population (Table 4). High genetic variation among regions and within populations has also been found in another congeneric species, *H*. *rhamnoides* ssp. *sinensis* based on cpSSR (30.9% for regions and 40.1% for within population) [61]. The significant genetic differentiation between the two regions separated by Mt. Everest indicates that the Himalayan mountians play a critical role in the genetic differentiation of populations. The PCA plot further confirms that the individuals of *H*. *tibetana* can be roughly divided into two groups, and reveals a geographical pattern with respect to Mt. Everest (Fig 4). Almost all individuals from the Nepalese populations (southern side of Mt. Everest) are grouped on the right side of PCA axis 1 whereas individuals from the Tibetan populations (northern side of Mt. Everest) are mostly positioned on the left side of PCA axis 1 (Fig 4), suggesting that gene flow between the southern and northern populations has been disrupted to a certain extent. Furthermore, the STRUCTURE result (Fig 5) reveals a pattern of geographically distinct north and south populations, strongly indicating a lack of gene flow across the Himalayan mountains in either direction. STRUCTURE genetic relations among populations also reflect the natural geographic locations of each population. The geographically close populations show low genetic differentiation, whereas the geographically distant populations show high differentiation. This is supported by an isolation-by-distance model for *H*. *tibetana* using the Mantel test (Fig 3), which detects a statistically significant positive correlation between genetic distance and geographical distance. In this case, it seems that population differentiation between the two regions is caused partially by distance, although the PCA, and especially the STRUCTURE analysis, indicate the absence of genetic exchange and restricted gene flow associated with the Himalayan mountains. Furthermore, it can be seen that low population differentiation and higher gene flow are found in populations from the same region, while high population differentiation and lower gene flow are found in populations from different regions (Table 5). All these characteristics of genetic structure of *H*. *tibetana* indicate strongly that there is restricted gene flow in *H*. *tibetana* in our study area. The physical barrier of the Himalayan mountains and isolation-by-distance has contributed to limiting gene flow in *H*. *tibetana* between the two regions.

**Table 5:** Matrix of pairwise population *F*st values (below) and *N*m (above) based on nuclear microsatellite data.

Population	NA	NB	NC	ND	TA	TB	TC
NA	—	6.160	3.681	5.252	0.370	0.258	0.976
NB	0.039	—	4.675	4.849	0.306	0.211	0.477
NC	0.064	0.051	—	6.760	0.324	0.238	0.545
ND	0.045	0.049	0.036	—	0.320	0.239	0.429
TA	0.404	0.450	0.435	0.439	—	2.875	3.500
TB	0.492	0.542	0.512	0.512	0.080	—	4.090
TC	0.293	0.344	0.314	0.368	0.067	0.058	—

*F*st, population differentiation; *Nm*, gene flow

Many studies have suggested that cpDNA is maternally inherited via seeds, not through pollen, in most angiosperms [74], [75], [76]. This characteristic may lead to very low gene flow and high differentiation among populations in many angiosperms [77]. High genetic variation (69.71%) (Table 4) between the southern and northern populations and high significant population differentiation among populations (*F*_ST_ = 0.731, *P* < 0.01) (Table 4) are found based on AMOVA of the cpDNA data of *H*. *tibetana*. This is most likely due to populations being separated by high mountains, namely by The Himalayan mountains, which results in limited seed flow of *H*. *tibetana* between our two regional populations. It has been reported that the seeds of *Hippophae* are dispersed by birds [78], and that large birds, such as the Bar-headed Goose (*Anser indicus*) often cross the Himalaya to spend winter in India or Nepal and summer in Tibet [79]. However, this seems not to be the case for seed flow in *H*. *tibetana*, because there are completely different haplotypes (Fig 1) on the two sides of Mt. Everest, indicating limited seed flow between the northern and southern sides of the Himalayan mountains. Similar landscape effects have also been observed in the Hengduan and Dabashan mountains, where high mountains might have been natural dispersal barriers for *Taxus wallichiana* [80].

Based on the cpDNA data, total genetic diversity (*H*T) of *H*. *tibetana* is high (0.668, Table 3) but within-population diversity (*H*s) is relatively low (0.331), which also indicates limited seed flow among populations. In contrast, there is less differentiation among populations from the same regions, because most of the haplotypes are shared among these populations, for example haplotype N1 occurs at high frequency in all the southern populations and haplotypes T1-T4 are distributed in all the northern populations.

### Phylogentic relationship and the haplotype distribution of *Hippophae tibetana*

According to our previous study, the northern valley of Mt. Everest was a refugium for *H*. *tibetana* during the Last Glacial Maximum (LGM) [30]. Compared with the three haplotype lineages (A, B, C) found in our previous study [30], two of the six haplotypes from this study, N1 and N2, are found for the first time in this study and are endemic to the southern region of Mt. Everest. Our northern haplotypes (T1, T2, T3, T4) correspond with haplotypes B14, B10, B13, and B3, in our previous study [30] (Fig 6). The divergence times of all these haplotypes could date back to between 0.15 and 1.53 million years ago according to our previous study [30] (Fig 6). In addition, the haplotype distribution in the present study has no common haplotype in the southern and northern populations of Mt. Everest, and SAMOVA analysis also further indicates a barrier that separates populations NA, NB, NC and ND from populations TA and TC. All these results suggest that populations of *H*. *tibetana* from the two regions may have remained isolated from each other during the Pleistocene. In other words, this pattern indicates a lack of *H*. *tibetana* seed flow across the Himalayan mountains at least since the Pleistocene. Interestingly, in the haplotype network, southern haplotypes N1 and N2 are not directly connected to any haplotypes from their nearest neighbors on the northern slope of Mt. Everest, but are connected to haplotype B16, which is found in the northwest part of Mt. Everest, about 85 km away from the southern valley of Mt. Everest.

Although the variable analysis applied above strongly supports our hypothesis that population differentiation in *H*. *tibetana* can be attributed to the restricted gene flow associated with significant geographic barriers created by the Himalayan mountains and isolation-by-distance, the possibility of environmental factors on population differentiation cannot be ruled out, because this may partially be responsible for the observed high population differentiation of *H*. *tibetana* between the two climatically distinct regions in our study area. The Himalaya is located in the inter-tropical convergence zone, and Mt. Everest is influenced by two dominant climate systems—the Mid-Latitude Westerlies and the South Asian Monsoon [81]. The climate on the northern slopes of Mt. Everest is semi-arid and cold, whereas on the southern slopes it is more humid, and correspondingly the vegetation on the southern slopes is mainly forest, whereas it is sparse open steppe with low shrubs and abundant grasses on the northern slopes [81]. Variable environmental factors can also contribute to population differentiation to some degree [41], thus the distinct climatic and vegetation types on the two sides of the Himalayan mountains may partially contribute to the current population differentiation on the two sides. Spatially variable habitat could probably further drive population differentiation of *H*. *tibetana* on the two slopes of Mt. Everest by a lack of gene flow. Therefore, it is worth further investigation to examine selective and adaptive mechanisms for *H*. *tibetana* population differentiations that may be caused by both natural geographic barriers and distinct environmental conditions.

In conclusion, both our genetic datasets support the hypothesis that the Himalayan mountains has served as a physical barrier to gene flow in *H*. *tibetana*. There are strong population differentiations between the southern and northern slopes of Mt. Everest. The lack of gene flow across the Himalayan mountains in either direction, possible increased genetic drift associated with distinct environmental conditions, vegetative reproduction, as well as the fragmented habitat of *H*. *tibetana* are probably mainly responsible for the genetic structure at both the landscape scale and the fine scale. However, it is difficult to separate the effects of these several putative factors on spatial variation in our current data. Further advanced research approaches (for example, high-throughput sequencing, comparative genomics) are needed in the future to explore the local adaptive mechanisms, especially selective and genetic drift of *H*. *tibetana* associated with distinct environments on the northern and southern slopes in the Himalayan area.
